# Explorative Screening of Bioactivities Generated by Plant-Based Proteins after In Vitro Static Gastrointestinal Digestion

**DOI:** 10.3390/nu12123746

**Published:** 2020-12-05

**Authors:** Camille Dugardin, Benoit Cudennec, Mélissa Tourret, Juliette Caron, Laetitia Guérin-Deremaux, Josette Behra-Miellet, Catherine Lefranc-Millot, Rozenn Ravallec

**Affiliations:** 1 UMR-T 1158, BioEcoAgro, University of Lille, F-59000 Lille, France; camille.dugardin@univ-lille.fr (C.D.); melissa.tourret@univ-lille.fr (M.T.); josette.behra@univ-lille.fr (J.B.-M.); 2 Nutrition and Health Research & Development, Roquette, F-62136 Lestrem, France; juliette.caron@roquette.com (J.C.); laetitia.guerin-deremaux@roquette.com (L.G.-D.); 3 Nutrition and Health Research & Development, Roquette, F-59110 La Madeleine, France; catherine.lefranc-millot@roquette.com

**Keywords:** plant-based protein digestion, biological activity, CCK, GLP-1, DPP-IV, opioid receptor, ACE, inflammation, IL-8, ROS production

## Abstract

The gastrointestinal digestion of food proteins can generate peptides with a wide range of biological activities. In this study, we screened various potential bioactivities generated by plant-based proteins. Whey protein as an animal protein reference, five grades of pea protein, two grades of wheat protein, and potato, fava bean, and oat proteins were submitted to in vitro SGID. They were then tested in vitro for several bioactivities including measures on: (1) energy homeostasis through their ability to modulate intestinal hormone secretion, to inhibit DPP-IV activity, and to interact with opioid receptors; (2) anti-hypertensive properties through their ability to inhibit ACE activity; (3) anti-inflammatory properties in Caco-2 cells; (4) antioxidant properties through their ability to inhibit production of reactive oxygen species (ROS). Protein intestinal digestions were able to stimulate intestinal hormone secretion by enteroendocrine cells, to inhibit DPP-IV and ACE activities, to bind opioid receptors, and surprisingly, to decrease production of ROS. Neither pro- nor anti-inflammatory effects have been highlighted and some proteins lost their pro-inflammatory potential after digestion. The best candidates were pea, potato, and fava bean proteins.

## 1. Introduction

According to the Food and Agriculture Organization (FAO), current trends predict a constant increase in worldwide population, reaching nearly 10 billion people in 2050. Moreover, the international recommended dietary allowance for protein is currently 0.8 g per kg of body weight [1]. As a consequence, global protein demand should increase to meet the nutritional needs of the growing population. Additionally, since 1961, worldwide protein consumption has increased by about 20 g per day and per person. In this context, it becomes essential to better characterize dietary proteins and to diversify their origins. Whereas the high proportion of animal-protein consumption in developed countries raises environmental concerns, relying on intensive livestock farming [2], plant-based proteins appear as a more sustainable alternative. Indeed, the environmental footprint of plant-based proteins is lower due to less greenhouse gas emission and water consumption, leading to a better protein delivery efficiency [3]. Moreover, the proportion of the population becoming vegan, vegetarian, or flexitarian is increasing, promoted by ecological and health concerns but also the ethical treatment of animals [4]. In parallel, according to the World Health Organization, more than 1.9 billion adults were overweight in 2016. Among them, more than 600 million were obese (13% of the worldwide adult population). Obesity is a major risk factor for the development of type 2 diabetes mellitus (T2DM), which is a metabolic disorder characterized by prolonged hyperglycemia, leading to complications like hypertension and associated cardiovascular diseases.

Beyond their nutritional role as the source of amino acids for protein synthesis, dietary proteins are known to be involved in a wide range of biological functions [5], particularly through the action of peptides generated during their digestion in the gastrointestinal tract. Indeed, during this enzymatic process, dietary proteins are first partially hydrolyzed by pepsin in the stomach, then by a cocktail of proteases (trypsin, chymotrypsin, carboxypeptidases) in the small intestine, and finally, by peptidases at the brush border membrane. This generates amino acids and bioactive peptides present in the intestinal lumen whose size, sequence, and structure vary and which modulate several physiological processes by acting locally and/or systemically [6,7,8]. Several health-related effects have been described for bioactive peptides coming from different sources, using in vitro tests or animal and human trials [9,10]. For instance, it has been shown that peptides generated by dietary protein digestion could play a beneficial role in the context of obesity and metabolic disorders through the peripheral regulation of food intake [11]. Peripheral regulation of short-term food intake by proteins involves the stimulation of intestinal hormone secretion following the recognition of nutrients, such as digested protein-derived peptides, at the apical level of the enteroendocrine cells. This intestinal “sensing” leads to the secretion of anorexigenic peptide hormones such as cholecystokinin (CCK) and glucagon-like peptide 1 (GLP-1) that act as peripheral signals leading to the end of food intake [12,13]. GLP-1 plays also a crucial role in glucose metabolism by its role as incretin [14], which is drastically reduced by the dipeptidyl peptidase-4 (DPP-IV) enzymatic action, removing dipeptides from their N-terminal side [15]. Hence, DPP-IV inhibitors are nowadays considered as an advanced class of agents for T2DM management [16] and in recent years, numerous studies evidenced “natural” DPP-IV inhibitory peptides from digested dietary proteins [17]. Food-derived peptides may also bind peripheral opioid receptors in the portal vein and indirectly induce satiety via gluconeogenesis [18,19]. Dietary protein-derived peptides are also promising in the context of hypertension and associated cardiovascular risk, particularly by their ability to inhibit the Angiotensin Converting Enzyme (ACE), a dipeptidyl carboxypeptidase which plays an important role in the regulation of blood pressure by cleaving angiotensin I to induce vasoconstriction [20]. Several in vitro and animal trials had also evidenced the attractive potential of food-derived peptides in the management of human inflammatory bowel disease (IBD) via their anti-inflammatory effect and antioxidant activities [21]. However, data describing the relationship existing between the source and quality of protein and their biological activities are limited and inconsistent. Moreover, studies comparing numerous protein sources are lacking.

The purpose of the present study was thus to investigate in vitro the ability of 10 dietary plant-based proteins to modulate (1) food intake and glucose homeostasis through CCK and GLP-1 secretion, DPP-IV activity, and opioid receptor binding; (2) blood pressure through ACE activity; (3) inflammation through interleukin-8 (IL-8) secretion; (4) oxidative stress through reactive oxygen species (ROS) production. To do that, an in vitro simulated gastrointestinal digestion (SGID) was firstly performed in order to reach study conditions closer to the physiological ones but also to highlight and compare the effect of non-digested and digested dietary proteins on these biological activities. The protein sources have been chosen for two reasons. On the one hand, some of these sources are rich in proteins (e.g., pea) and thus, interesting for human consumption. On the other hand, some of these sources generate a lot of protein by-products (e.g., potato), which could be valorized.

## 2. Materials and Methods

### 2.1. Protein Samples

Ten plant-based protein samples were provided by Roquette (Table 1):

- Four grades of pea protein (PeaP1, PeaP2, PeaP3, and PeaP4);
- Hydrolyzed pea protein (HPeaP);
- Two grades of wheat protein (WP1 and WP2);
- Potato protein (PP);
- Fava bean protein (FBP);
- Oat protein (OP).

We performed two sets of analysis: the first one including PeaP1, HPeaP, WP1, WP2, and PP; the second one including PeaP2, PeaP3, PeaP4, FBP, and OP. Whey protein (WhP) was used as an animal protein reference and common control in each set of analysis.

### 2.2. Materials

Porcine pepsin (EC 3.4.23.1, from porcine gastric mucosa, 3850 U mg^−1^ protein), pancreatin from porcine pancreas (4 × USP specifications (5.46 U mg^−1^ based on trypsin activity), EC 232-468-9), Dipeptidyl Peptidase IV (DPP-IV from porcine kidney, EC 3.4.14.5, ≥10 U mg^−1^ protein), Gly-Pro-p-nitroanilide hydrochloride, Angiotensin Converting Enzyme (ACE from rabbit lung, EC 3.4.15.1), and all other reagents were purchased from Sigma-Aldrich (Sigma-Aldrich, Steinheim, Germany). The Active Glucagon-Like Peptide RIA kit (Cat.# GLP1A-35HK) was purchased from Merck Millipore (Merck-Millipore, Darmstadt, Germany). The Gastrin/CCK kit was purchased from CisBio (CisBio, Saclay, France). Human IL-8/CXCL8 Quantikine ELISA Kit (D8000C) was purchased from R&D Systems (R&D Sytems, Minneapolis, MN, USA).

### 2.3. In Vitro Simulated Gastrointestinal Digestion (SGID)

In vitro simulated gastrointestinal digestion (SGID) was performed as previously described [22]. The first three steps of digestion were simulated (mouth, stomach, and duodenum) and three fluids were prepared to mimic the physiological conditions of each step. The composition of each fluid was described in Table 2 and the pH of the solutions was adjusted to physiologically relevant values using NaOH (5 M) and HCl (5 M) solutions. The whole digestion process was performed in a 200 mL reactor controlled at 37 °C under constant stirring with a magnetic stirrer over 240 min. Then, 2 g of native protein sample (dry weight) was added to the reactor and solubilized in 16 mL salivary fluids at pH 6.8 (125 mg mL^−1^ dry matter). After 5 min, sampling of 4 mL was performed at the end of the salivary step (salivary sample). Next, 24 mL of gastric fluids containing pepsin were added after saliva sampling at an Enzyme: Substrate (E:S) ratio of 1:40 (*w*/*w*) and the pH of the solution was adjusted (2.5–3.0). After 2 h, sampling of 16 mL was performed at the end of the gastric step (gastric digestion—41.67 mg mL^−1^ dry matter). A total of 36 mL of intestinal fluids (24 mL of duodenal juice and 12 mL of bile juice) containing pancreatin at a E:S ratio of 1:50 (*w*/*w*) and 4 mL of 1 M NaHCO_3_ were then added to the batch and the pH of the solution was adjusted to 7. Intestinal digestion was carried out again over 2 h and sampling of 60 mL was performed at the end of the intestinal step (intestinal digestion). Intestinal digestion concentration reached 13.89 mg mL^−1^ dry matter. Once heated at 95 °C for 10 min to assure enzyme denaturation without peptide damage, all samples were centrifuged at 13,400× *g* for 10 min at room temperature. Supernatants were collected and frozen for further analysis.

Two sets of analysis were performed: the first one including PeaP1, HPeaP, WP1, WP2, PP, and WhP; the second one PeaP2, PeaP3, PeaP4, FBP, OP, and WhP. SGID was achieved in triplicates on each of the 10 protein samples provided by Roquette. As the reproducibility was demonstrated, SGID on the animal protein reference WhP was performed in triplicate for the first set of analysis and only once for the second set of analysis.

A blank SGID (without any protein sample but with digestive enzymes) was also achieved in order to exclude any activity due to enzyme autolysis in all further analysis.

### 2.4. Solubility Tests

The solubility degree of each protein sample was evaluated at each step of the SGID process (Table 3). To do that, dry matter percentage (% DM) of each sample before and after centrifugation at 13,400× *g* for 10 min at room temperature was assessed using a desiccator. The solubility degree was calculated by the ratio between % DM after centrifugation and % DM before centrifugation. The solubility degree was then applied to theoretical dry matter concentration to predict the real dry matter concentration of each protein sample at each step of the SGID process. All further analyses were based on this predicted dry matter concentration.

### 2.5. Size Exclusion Chromatography

To evaluate SGID triplicate reproducibility, size exclusion chromatography was used to analyze and compare the peptide molecular weight distribution of the digested samples (intestinal digestion). Separation was performed at room temperature using a Superdex Peptide 10/300 GL column under isocratic elution at a flow rate of 0.5 mL min^−1^ with a 30% acetonitrile, 0.1% TFA solvent. Absorbance was monitored at 214 nm using an AKTA purifier protein purification system (GE Healthcare, Amersham, UK). The column was calibrated using standard peptides (Albumin, 60 kDa; Cytochrome C, 12,400 Da; Aprotinin, 6500 Da; Vitamin B12, 1355 Da; Glutathione, 307 Da). The relationship between the Log of molecular weight standard peptides and the elution volume was used to perform the molecular weight profiles of the samples.

### 2.6. Bioactivity Tests

All further analyses were performed on each protein sample, at least before and after SGID.

#### 2.6.1. CCK and GLP-1 Secretion Study

The CCK and GLP-1 secretion study was performed as previously described [22]. STC-1 cells were seeded into 24-well culture plates at a density of 30 × 10^3^ per well and allowed to reach 60–80% confluence. Then, cells were washed and incubated with 3 different concentrations (0.2%, 0.5%, 1% *w*/*v*; dose response in triplicates) of protein samples before (salivary sample) and after SGID (intestinal digestion). Dilutions were made in incubation buffer (4.5 mM KCl, 1.2 mM CaCl_2_, 1.2 mM MgCl_2_, 140 mM NaCl, and 20 mM Hepes-Tris, pH 7.4). Control wells contained only incubation buffer. After 2 h incubation at 37 °C in 5% CO_2_ atmosphere, supernatants were collected on ice and centrifuged (2000× *g* for 7 min). The supernatants were frozen and stored at −20 °C for further hormone concentration determination. Secreted CCK and GLP-1 concentration (pM) in culture media of STC-1 cells was assayed using commercial RIA kits: CCK (CisBio, Saclay, France) and GLP-1 (Merck-Millipore, Darmstadt, Germany).

#### 2.6.2. DPP-IV Inhibition Assay

The DPP-IV inhibitory activity of the protein samples before (salivary sample), during (gastric digestion), and after SGID (intestinal digestion) was assayed according to the method described by Lacroix et al. [23] with some modifications. Concisely, 25 µL of each sample at concentrations ranging from 1.21 to 13.89 mg mL^−1^ (dose response of 4 concentrations in triplicates) were pre-incubated with 75 µL of Tris/HCl buffer (100 mM, pH 8.0) and 25 µL of DPP-IV (0.018 U mL^−1^) at 37 °C for 5 min in a 96-well plate. For control purposes, the sample was replaced with Tris/HCl buffer (100 mM, pH 8.0). Then, the reaction was initiated by the addition of 50 µL of Gly-Pro-p-nitroanilide (1 mM). All the samples and reagents were diluted in Tris/HCl buffer. The plate was then incubated at 37 °C for 1 h, and the absorbance of the released p-nitroanilide at 405 nm at 2 min intervals assayed with a microplate reader (ELX808, Biotek, Winoosky, VT, USA). The concentration of the sample required to obtain 50% inhibition of the DPP-IV activity (IC_50_) was determined by plotting the % DPP-IV inhibition as a function of the sample’s final concentration natural logarithm. IC_50_ were expressed in mg mL^−1^ dry matter.

#### 2.6.3. Opioid Receptor Binding Assay

The potential binding on opioid receptors of the samples before (salivary sample) and after SGID (intestinal digestion) was assessed in a radiobinding competition test with ^3^H-naloxone as the tritiated specific ligand on rat brain membrane preparation, described by Garreau et al. [24] with some modifications. Briefly, rat brains were homogenized in a buffer (50 mM Tris-HCl, 240 mM sucrose, 5 mM MgCl_2_, and 2 mM EDTA) and centrifuged at 1000× *g* for 5 min at 4 °C. Supernatants were then centrifuged at 30,000× *g* for 30 min at 4 °C and the resulting pellets were resolubilized in the homogenization buffer. A protein assay was performed with the Sigma QuantiPro BCA Assay Kit. Serial dilution of native protein samples (salivary sample) and their SGID products (intestinal digestion) ranging from 0.6 to 36 mg mL^−1^ (dose response of 6 concentrations) or naloxone as positive control were realized in 50 mM Tris-HCl buffer pH 7.4 supplemented with 2% bovine serum albumin and were incubated in 0.9 mg mL^−1^ of rat brain membrane preparation with the addition of protease inhibitors, bestatin (10 µM) and thiorphan (0.1 µM), and 1 nM of ^3^H-naloxone for 30 min at 25 °C. Non-specific binding was determined by adding an excess of naloxone (5 µM) instead of the sample or naloxone dilutions. All conditions were run in duplicates. At the end of the incubation, separation of bound and unbound samples was performed by vacuum filtration through glass microfiber GF/B filters. Filters were inserted into scintillation vials, 3 mL Optiphase HiSafe 2 scintillation liquid was added, and radioactivity was counted on a beta-counter (Hidex 300 SL, Sciencetec, Villebon-sur-Yvette, France). Non-specific binding was subtracted from all values and specific binding was expressed as percentage of total specific binding (cpm). ED_50_ (effective dose 50%) are determined by non-linear regression analysis. ED_50_ is the dose of sample that removes 50% of ^3^H-naloxone maximal binding to opioid receptors in order to replace it.

#### 2.6.4. ACE Inhibition Assay

The ACE inhibitory activity of the different native protein samples (comparable to salivary sample) as compared to their SGID products (gastric and intestinal digestions) was assayed according to the protocol described by Sentandreu and Toldra [25]. Concisely, 50 µL of diluted samples at concentrations ranging from 0.30 to 13.89 mg mL^−1^ (dose response of 4 concentrations in duplicates) were pre-incubated with 50 µL of ACE working solution (50 mU mL^−1^ enzyme activity) in a 96-well plate. The plate was then carefully shaken for a few seconds and incubated at 37 °C for 10 min. The enzyme reaction was started by adding 200 µL of fluorescent substrate working solution (o-aminobenzoylglycyl-p-nitro-L-phenylalanyl-L-proline Abz-Gly-Phe(NO_2_)-Pro at 0.45 mM). The plate was again carefully shaken for a few seconds and incubated at 37 °C. Fluorescence was measured after 30 min at excitation wavelength 365 nm and emission wavelength 415 nm using a Xenius XC spectrofluorometer. The concentration of the sample required to obtain 50% inhibition of the ACE activity (IC_50_) was determined by plotting the % ACE inhibition as a function of the sample’s final concentration natural logarithm. IC_50_ were expressed in µg mL^−1^ dry matter.

#### 2.6.5. IL-8 Secretion Assay

Caco-2 cells were seeded into 24-well culture plates at a density of 4 × 10^4^ cells per well and allowed to reach confluence and differentiation (2 weeks). Differentiated Caco-2 cells were submitted or not during 24 h to inflammatory stimuli with LPS (lipopolysaccharides, 20 µg mL^−1^, Sigma-Aldrich). Then, cells were incubated with 3 different concentrations (0.1%, 0.25%, 0.5% (*w*/*v*); dose response in triplicates) of native protein samples (salivary sample) compared to their SGID products (intestinal digestion) diluted in incubation buffer (4.5 mM KCl, 1.2 mM CaCl_2_, 1.2 mM MgCl_2_, 140 mM NaCl, and 20 mM Hepes-Tris, pH 7.4). Control wells contained only incubation buffer. After 24h incubation at 37 °C in 5% CO_2_ atmosphere, supernatants were collected on ice and centrifuged (2000× *g* for 7 min). The supernatants were frozen and stored at −20 °C for further cytokine concentration determination. Secreted IL-8 in culture media of Caco-2 cells was evaluated using commercial ELISA kits.

#### 2.6.6. Antioxidant Assays

##### Superoxide Anion Assay

Concentration of O_2_^−^ was estimated using an acellular method, adapted from that reported by Aruoma et al. [26]. Briefly, the superoxide anion (7 to 10 nmol mL^−1^) was produced in Hank’s HEPES buffer (pH 7.42) using a xanthin (0.1 mM)—xanthin oxidase (50 mU mL^−1^) system. This O_2_^−^ quantity was then incubated with different quantities of native protein samples and their SGID products from 1.25 to 5 mg mL^−1^ (dose response of 3 concentrations in triplicates) for 15 min at 25 °C with equine ferricytochrome c (FerC) 0.017 mM. Controls without the sample and with only O_2_^−^ were also incubated. FerC (orange) was reduced to ferricytochrome c (pink) by the remaining superoxide anion radicals and the absorbance was measured by spectrophotometry at 550 nm. Concentrations of O_2_^−^ were determined thanks to the ferricytochrome c extinction coefficient (21.1 × 10^3^ L mol^−1^ cm^−1^) and the Beer-Lambert law and were expressed in nmol.mL^−1^. The same experiments were also performed on the salivary sample and the intestinal digestion of blank SGID (SGID only with enzymes), which induce a decrease in O_2_^−^ production. To avoid any interference, IC_50_ values for the 11 protein samples (salivary sample and intestinal digestion) were calculated taking into account the individual effects of the blank SGID samples.

##### Hydrogen Peroxide Assay

Concentration of H_2_O_2_ was estimated using an acellular method, adapted from that reported by Thurman et al. [27]. Briefly, about 10 nmol.mL^−1^ of H_2_O_2_ was incubated with different quantities of native protein samples and their SGID products from 1.25 to 5 mg mL^−1^ (dose response of 3 concentrations in triplicates) for 15 min at room temperature in Hank’s HEPES (HH) buffer (pH 7.42). Controls without H_2_O_2_ (replaced by HH) were also incubated for each sample dilution. After adding 40 µL of 1N HNO_3_, 200 µL of 10 M Iron (II) ammonium sulfate, and 100 µL of 2.5 M KSCN (potassium thiocyanate), the tubes were mixed and the absorbance, reflecting the ferric thiocyanate red complexes, was measured by spectrophotometry at 480 nm. Concentrations of H_2_O_2_ in the samples were determined by using a standard curve ranging from 2.5 to 20 nmol.mL^−1^. The same experiments were also performed on the salivary sample and the intestinal digestion of blank SGID (SGID only with enzymes) which induce a decrease in H_2_O_2_ production. To avoid any interference, IC_50_ values for the 11 protein samples (salivary sample and intestinal digestion) were calculated taking into account the individual effects of the blank SGID samples.

##### Hydroxyl Radical Assay

Concentration of HO was estimated using an acellular method, adapted from that reported by Halliwell et al. [28]. In this method, HO^.^ was produced from H_2_O_2_ (10 to 13 nmol mL^−1^) in a KH_2_PO_4_ 20mM buffer (pH 7.4) by the Fenton reaction, with EDTA-Fe^2+^ (FeCl_3_ 100 µM and EDTA 104 µM) and ascorbic acid (100 µM). HO^.^ was then incubated with different quantities of native protein samples and their SGID products from 1.25 to 5 mg mL^−1^ (dose response of 3 concentrations in triplicates) and deoxyribose (3 mM) for 30 min at 37 °C. To highlight deoxyribose degradation, samples were heated for 20 min to generate malondialdehyde and incubated with thiobarbituric acid (14 mM) in an acidic medium (trichloroacetic acid, 147 mM) to yield a pink chromogen. The absorbance was then measured by spectrophotometry at 532 nm. Concentrations of HO· in the samples (nmol mL^−1^) were determined by using a standard curve. The same experiments were also performed on the salivary sample and the intestinal digestion of blank SGID (SGID only with enzymes), which induce a decrease in HO production. To avoid any interference, IC_50_ values for the 11 protein samples (salivary sample and intestinal digestion) were calculated taking into account the individual effects of the blank SGID samples.

## 3. Results and Discussion

Whey protein (WhP) is used as an animal-based protein reference in this study, since dairy proteins and peptides have already been particularly explored in those contexts [29,30,31].

### 3.1. Good Reproducibility of SGID Process and Peptide Generation

The shapes of replicate chromatograms for each protein intestinal digestion (Supplementary Figures S1 to S11) are comparable, indicating that the SGID process is reproducible. Moreover, the shapes of chromatograms are not different when comparing SGID performed on the control WhP (Supplementary Figure S11) during the first (WhP-1, WhP-2, and WhP-3) and during the second study phases (WhP-4), indicating that both are comparable. Finally, the chromatogram obtained for the intestinal digestion of blank SGID (Supplementary Figure S12) and its comparison with all other chromatograms (Supplementary Figures S1 to S11) seem to indicate that the peptide profiles of intestinal digestions for each protein sample are only very slightly contaminated by non-specific peptides generated by the hydrolysis and autolysis of the digestive enzymes themselves (pepsin and pancreatin) in this SGID protocol.

**Figure 1:**
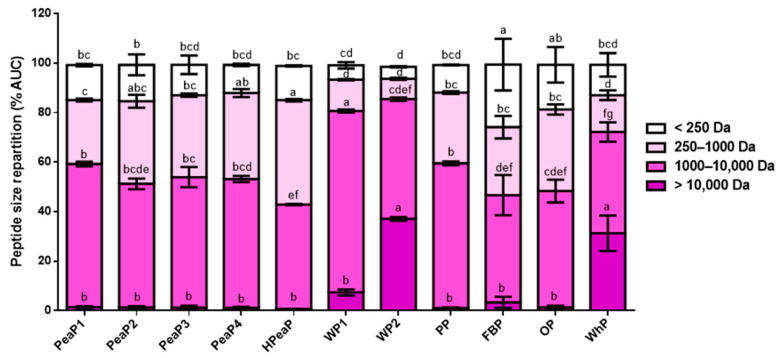
Peptide size repartition after SGID of the different protein samples (intestinal digestion). Values are reported as the mean from triplicate determinations with SD and are expressed in % chromatogram AUC. Means for each peptide size class not sharing any common letter (a–g) are significantly different according to two-way ANOVA procedure followed by Tukey’s test. PeaP1–PeaP4 (4 grades of pea protein), HPeaP (hydrolyzed pea protein), WP1–WP2 (2 grades of wheat protein), PP (potato protein), FBP (fava bean protein), OP (oat protein) and WhP (Whey protein).

Moreover, when comparing peptide size repartition in each protein intestinal digestion (Figure 1), SGID of WP1, WP2, and WhP generates longer peptides (>10,000 Da for WP2 and WhP and between 1000 and 10,000 Da for WP1). In contrast, SGID of HPeaP, FBP, and OP generates smaller peptides (between 250 and 1000 Da for HPeaP and <250 Da for FBP and OP). Prior hydrolysis of pea protein (HPeaP) thus leads to smaller peptides than the four grades of pea proteins (PeaP1, PeaP2, PeaP3, and PeaP4), whose peptide size repartition is comparable.

These results indicate that the SGID of the different protein samples generates peptides with different sizes, sequences, and structures. This could explain the differences observed in terms of bioactivities.

### 3.2. Dose-Dependent Increase in CCK and GLP-1 Secretion in Response to Intestinal Digestions (after SGID)

Digested bovine hemoglobin is used as a positive control since its important effect on intestinal hormone secretion has already been demonstrated [22].

**Figure 2:**
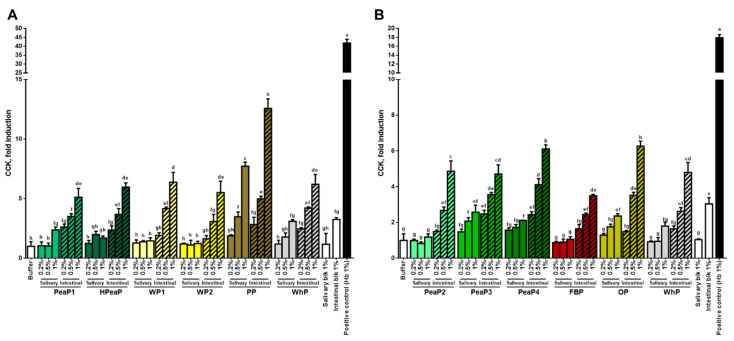
STC-1 cells were incubated for 2 h with different final concentrations (0.2, 0.5, and 1% dry matter) of the protein samples of the first (**A**) and the second phase (**B**), before (salivary sample) and after SGID (intestinal digestion). CCK levels were determined by RIA and expressed as % of control (buffer) levels. Data are expressed in mean (*n* = 3) ± SD. Means without a common letter (a–h) are different (*p* < 0.05) according to one-way ANOVA procedure followed by Tukey’s test. PeaP1–PeaP4 (4 grades of pea protein), HPeaP (hydrolyzed pea protein), WP1–WP2 (2 grades of wheat protein), PP (potato protein), FBP (fava bean protein), OP (oat protein) and WhP (Whey protein).

**Figure 3:**
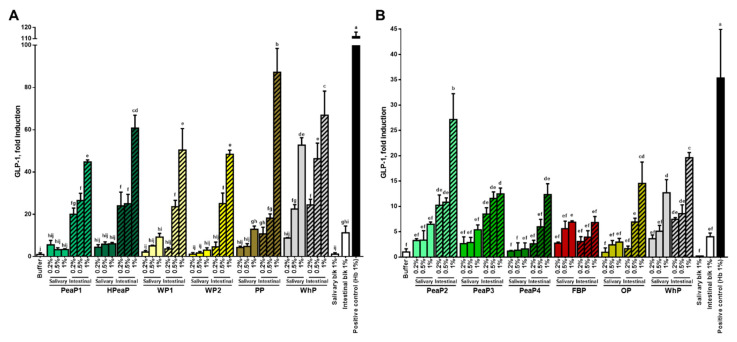
STC-1 cells were incubated for 2 h with different final concentrations (0.2, 0.5, and 1% dry matter) of the protein samples of the first (**A**) and the second phase (**B**), before (salivary sample) and after SGID (intestinal digestion). GLP-1 levels were determined by RIA and expressed as % of control (buffer) levels. Data are expressed in mean (*n* = 3) ± SD. Means without a common letter (a–j) are different (*p* < 0.05) according to one-way ANOVA procedure followed by Tukey’s test. PeaP1–PeaP4 (4 grades of pea protein), HPeaP (hydrolyzed pea protein), WP1–WP2 (2 grades of wheat protein), PP (potato protein), FBP (fava bean protein), OP (oat protein) and WhP (Whey protein).

For all the samples and conditions tested, the intestinal digestion (after SGID) leads to a better induction of CCK (Figure 2) and GLP-1 (Figure 3) secretion by STC-1 cells than the salivary sample (before SGID), even if some studies indicate that intact proteins can be more potent than hydrolysates [32,33]. Moreover, the incubation of STC-1 cells with the different intestinal digestions increases CCK and GLP-1 secretion in a dose-dependent manner.

Furthermore, as a reference, the levels of CCK secretion induced by WhP after SGID during the first phase (Figure 2A) and during the second study phase (Figure 2B) are comparable; indeed, the incubation of STC-1 cells with the intestinal digestion of WhP (final concentration 1%) leads to a CCK secretion of 63.80 ± 8.38 pM (or ~6-fold increase compared to the buffer control) during the first phase (Figure 2A) and of 60.17 ± 7.07 pM (or ~5-fold increase compared to the buffer control) during the second phase (Figure 2B). This indicates that the two phases of the CCK secretion study are comparable.

However, when comparing the impact of SGID performed on WhP during the first phase (Figure 3A) and during the second study phase (Figure 3B) on GLP-1 secretion, there are some differences. Indeed, the incubation of STC-1 cells with the intestinal digestion of WhP (final concentration 1%) leads to a ~60-fold increase in GLP-1 secretion compared to the buffer control during the first phase (Figure 3A), whereas it reaches a ~20-fold increase in GLP-1 secretion compared to the buffer control during the second phase (Figure 3B). In any case, this 3-fold difference between the first and the second phases can also be detected for other samples which are common to the two phases of the study (Figure 3) like the intestinal digestion obtained after blank SGID (intestinal blk 1%) and the positive control (bovine hemoglobin Hb 1%), leading to the conclusion that STC-1 cells were not as responsive regarding GLP-1 secretion in the second study phase as in the first one. Nevertheless, the two phases of the study can be compared based on GLP-1 secretion induced by WhP.

The incubation of STC-1 cells with the intestinal digestion obtained after blank SGID (intestinal blk 1%) leads to a ~3-fold increase in CCK secretion (Figure 2) and also an increase in GLP-1 secretion compared to the buffer control (Figure 3). However, all intestinal digestions tested at this concentration (1%) lead to a higher CCK (between ~5- and ~12-fold) and GLP-1 secretion than the intestinal blank. The compound inducing the best CCK secretion is PP (Figure 2A) and the ones inducing the best GLP-1 secretion are PP (Figure 3A) and PeaP2 (Figure 3B).

Numerous studies using different in vitro and in vivo models have already demonstrated the effect of hydrolysates obtained from various protein sources like milk [31], meat [22], fish and crustaceans [34,35], and plants [36] on gut hormone secretion. Nevertheless, only few studies aim at comparing the effects of several protein sources. One of them has compared the effect of pea, potato, soy, whey, and casein protein hydrolysates on CCK secretion using STC-1 cells but no significant differences in impact were observed between the different hydrolysates [37]. Another study has shown that pea and wheat proteins were the most potent stimulators of CCK and GLP-1 release from human duodenal biopsies in Ussing Chambers compared to animal proteins like ovomucoid, egg, and codfish proteins [38]. Recently, it has been highlighted that wheat gluten hydrolysate more potently stimulated GLP-1 secretion by GLUTag cells than lactalbumin enzymatic hydrolysate [39].

The present study allows the direct comparison, in the same conditions, between different plant-based proteins and whey protein and reveal that all proteins tested, once digested, stimulate CCK and GLP-1 secretion by STC-1 cells, potato and pea proteins being the most potent stimulators.

### 3.3. Good Inhibition of DPP-IV Activity by Intestinal Digestions (after SGID)

For all proteins, the intestinal digestion (after SGID) leads to better inhibition of DPP-IV activity (Table 4) than the gastric digestion (during SGID) and salivary sample (before SGID). The results indicate that the SGID of these 11 protein samples generates potential bioactive peptides, which are effective DPP-IV activity inhibitors since IC_50_ values for intestinal digestions are between ~0.5 and 2 mg mL^−1^ (Table 4), the best ones being HPeaP, FBP, and OP with, respectively, IC_50_ values of 0.79 ± 0.08, 0.54 ± 0.03, and 0.83 ± 0.10 mg mL^−1^ (intestinal digestion). Interestingly, the SGID of these three protein samples is shown to generate smaller peptides (Figure 1). Moreover, whereas SGID of the WP2 is shown to generate longer peptides (Figure 1), its IC_50_ value for DPP-IV activity is the highest in this study (2.03 ± 0.30 mg mL^−1^ (intestinal digestion)), suggesting peptide size is important for this activity. Then, the same experiment was performed on the blank SGID (only with enzymes) and no DPP-IV inhibitory activity was detected in this intestinal digestion.

IC_50_ values for DPP-IV activity obtained in this study are really remarkable, since IC_50_ values of various digestions are known to generally range from 1 to 5 mg mL^−1^. As an example, in the literature, IC_50_ values for digested bovine hemoglobin and cuttlefish are, respectively, 1.62 and 2.15 mg mL^−1^ [40]; for digested milk protein isolate, it is 0.65 ± 0.06 mg mL^−1^ [41]; for digested hemp, pea, rice, and soy protein isolate, it is between 1.85  ±  0.34 and 4.50  ±  0.55 mg mL^−1^ [42]. A recent review has listed dietary protein-derived hydrolysates displaying IC_50_ lower than 1 mg mL^−1^ [17], in which digested oat flour is reported (0.99 mg mL^−1^ [43]).

The present study allows highlighting of the potency of different digested plant-based proteins as DPP-IV inhibitors, pea, fava bean, and oat proteins being the most effective. These results could be confirmed using more biological relevant models and cell lines expressing DPP-IV-like Caco-2 cells [40]. Nevertheless, the impact of the brush barrier peptidases activity on the DPP-IV inhibitory peptides and their passage through the brush border membrane has yet to be investigated to evaluate their potential to inhibit plasma DPP-IV activity.

### 3.4. Good Potential to Bind Opioid Receptors by Protein Samples

All samples tested (salivary sample (before SGID) and intestinal digestion (after SGID)) can potentially bind opioid receptors (Table 5). Moreover, SGID does not always lead to an improvement of the potential binding of the sample on opioid receptors, since ED_50_ values for salivary sample and intestinal digestion are comparable, like for PeaP2, PeaP3, HPeaP, and PP. Nevertheless, the SGID process improves opioid receptor binding for PeaP1, PeaP4, WP1, WP2, OP, and WhP. In one case, SGID process decreases opioid receptor binding (FBP).

The samples having the highest affinity for opioid receptors are PeaP2 and PeaP3 with, respectively, an ED_50_ value of 4.98 and 4.63 mg.mL^−1^ before SGID (salivary sample) and 4.23 and 2.44 mg.mL^−1^ after SGID (intestinal digestion) (Table 5). The same experiment was performed on the blank SGID (only with enzymes) and no binding on opioid receptors was detected in this sample.

A lot of peptides, derived from food proteins like casein and lactalbumin (milk), glutenin and gliadin (wheat), RuBisCo (spinach), or β-conglycinin (soybean), are known to be recognized by opioid receptors, and display opioid-like molecular and physiological activities [44].

The present study allows us to highlight new proteins, digested or intact, able to bind opioid receptors, pea proteins being the most effective. Nevertheless, the passage of protein/peptide sequences, through the brush border membrane, has yet to be investigated as it has been shown for hemorphin peptides generated by SGID of bovine hemoglobin [45], to further evaluate their potential to bind peripheral opioid receptors in the portal vein, leading to the regulation of food intake. Moreover, food-derived opioid peptides could also reach the brain and thus, be implicated in several other biological functions, as they were able to cross the blood-brain barrier in vitro [45].

### 3.5. Good Inhibition of ACE Activity by Intestinal Digestions (after SGID)

The SGID of the 11 protein samples generates potential bioactive peptides which are good ACE activity inhibitors, since IC_50_ values for intestinal digestions are between ~30 and 300 µg mL^−1^ (Table 6), the best ones being PeaP4 with an IC_50_ value of 29.54 ± 34.98 µg mL^−1^ (intestinal digestion), WP1 with an IC_50_ value of 63.82 ± 20.18 µg mL^−1^ (intestinal digestion), PP with an IC_50_ value of 85.23 ± 19.24 µg mL^−1^ (intestinal digestion), and FBP with an IC_50_ value of 52.02 ± 43.06 µg mL^−1^ (intestinal digestion). Moreover, the same experiment was performed on the blank SGID (only with enzymes) and no ACE inhibitory activity was detected in this sample.

Peptides with ACE-inhibiting activity have been detected in various sources including milk, eggs, meat, plants, and fish [46,47]. IC_50_ values for ACE activity obtained in this study are satisfying since they are comparable with other studies performed on peptides from different sources [48]. A study has compared the ACE-inhibitory activity of the hydrolysates obtained by the pepsin digestion of some plant-based proteins, such as chickpea, common bean, lentil, lupin, pea, and soybean, the most efficient being soybean and lupin with respective IC_50_ values of 224 and 226 µg mL^−1^ [49]. Another study has identified ACE-inhibiting peptides in enzymatic hydrolysates of plant-based proteins such as rice, soy, pea, and wheat, with IC_50_ values ranging from 27 to 39 µg mL^−1^ [50].

The present study allows highlighting of the potency of different digested plant-based proteins as ACE inhibitors, pea, wheat, potato, and fava bean being the most effective. These results should be confirmed using, for instance, cell models expressing ACE.

### 3.6. No Effect on Pro-Inflammatory Cytokine Secretion by Protein Samples

**Figure 4:**
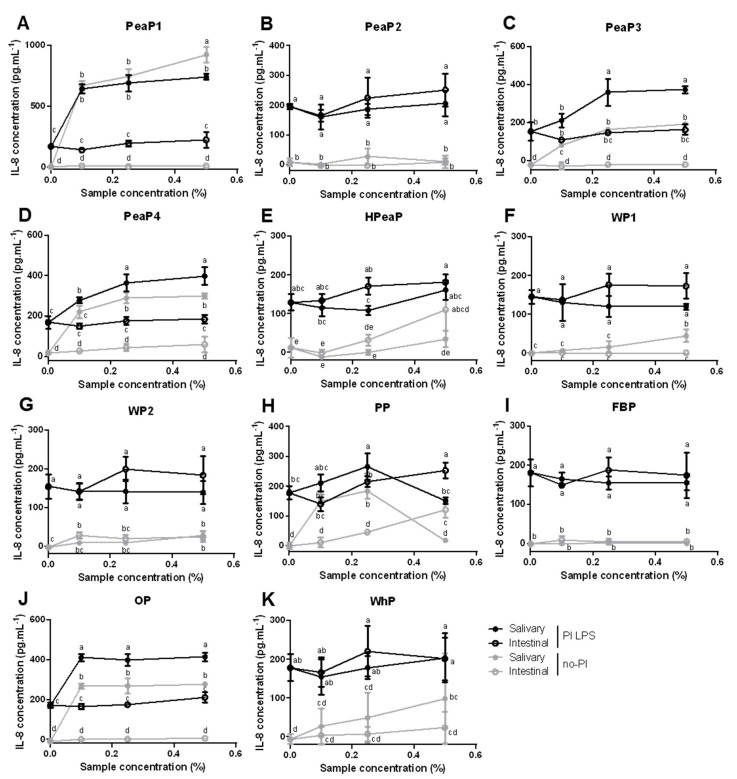
Differentiated Caco-2 cells were pre-incubated (PI-black) or not (no-PI-grey) with 20 µg.mL^−1^ LPS for 24h and then, incubated with different final concentrations (0.1, 0.25, and 0.5% dry matter) of the 11 protein samples, before (salivary sample—filled circles) and after SGID (intestinal digestion—empty circles) for 24h. IL-8 levels were determined by ELISA and expressed in pg mL^−1^. Data are expressed in mean (*n* = 3) ± SD. Means without a common letter (a–e) are different (*p* < 0.05) according to two-way ANOVA procedure followed by Tukey’s test. (**A**–**D**): PeaP1–PeaP4 (4 grades of pea protein), (**E**): HPeaP (hydrolyzed pea protein), (**F**,**G**): WP1–WP2 (2 grades of wheat protein), (**H**): PP (potato protein), (**I**): FBP (fava bean protein), (**J**): OP (oat protein) and (**K**): WhP (Whey protein).

Incubation with the salivary sample (before SGID) of PeaP1, PeaP3, PeaP4, and OP significantly increases IL-8 secretion, with or without pre-inflammation with LPS, suggesting a pro-inflammatory effect of these protein samples (Figure 4). However, this increase is not observed following incubation of these four protein samples after SGID, suggesting that they are no more pro-inflammatory after digestion. This can be explained by the destruction of certain inflammatory peptide structures by digestive enzymes during SGID. A similar but slighter effect of the SGID can also be observed for WP1 and WhP without pre-inflammation with LPS (Figure 4).

Moreover, the results indicate that incubation with intestinal digestions of the 11 protein samples does not lead to significant changes in IL-8 secretion by Caco-2 cells pre-inflamed or not with LPS and thus, does not highlight any pro- or anti-inflammatory properties of the samples on cytokine secretion (Figure 4).

Peptides with anti-inflammatory activity have been detected in various sources including milk casein, wheat gluten, egg ovotransferrin, and soybean protein [51]. The present study does not allow us to highlight the anti-inflammatory properties of the different proteins tested, digested or intact, on LPS-induced IL-8 secretion by Caco-2 cells. However, some further analyses including the use of TNF-α [52] or IL-1β [53] as inflammation inducers, other cytokines, or prostaglandins and cell models are needed to evaluate their potential role in inflammation processes to complete the present study. Moreover, it would be interesting to use a model of co-culture Caco-2/immune cells.

### 3.7. Antioxidant Properties of Protein Samples (after SGID)

#### 3.7.1. Superoxide Anion Assay

Only salivary samples of PeaP1, WP2, PP, and OP decrease O_2_^−^ production (Table 7) but this antioxidant activity is lost after SGID (intestinal digestion), except for PP which is still able to decrease O_2_^−^ production.

#### 3.7.2. Hydrogen Peroxide Assay

Most of the samples (salivary samples and intestinal digestions) decrease H_2_O_2_ production (Table 8). PeaP1, PeaP2, HPeaP, PP, and FBP exhibit a good IC_50_ value before and after SGID, while PeaP3 and OP inhibit more H_2_O_2_ production before than after SGID. Finally, WhP inhibits more H_2_O_2_ production after than before SGID.

#### 3.7.3. Hydroxyl Radical Assay

All samples (salivary samples and intestinal digestions) decrease HO^.^ production (Table 9). Most of the samples, such as PeaP1, PeaP2, PeaP3, PeaP4, HPeaP, WP2, PP, FBP, and OP, exhibit a good IC_50_ value before and after SGID, while WP1 and WhP inhibit more HO^.^ production after than before SGID.

PP is the only sample that inhibits O_2_^−^, H_2_O_2_, and HO^.^ production before (salivary sample) and after (intestinal digestion) SGID. All samples are able to inhibit HO^.^ production after SGID (intestinal digestions) and most of them are able to inhibit H_2_O_2_ production too after SGID (PeaP1, PeaP2, PeaP3, HPeaP, PP, FBP, OP, and WhP).

The composition of the samples, for instance, the release during digestion of small peptides with a thiol function could explain a powerful antioxidant effect. Food protein hydrolysates and peptides with antioxidant activity have been detected in various sources [54]. However, the antioxidant properties are determined mostly based on in vitro ability to scavenge free radicals, reduce ferric iron to ferrous, bind metals, and inhibit lipid oxidation.

The present study allows highlighting of, for the first time, the antioxidant properties of the different plant-based proteins, digested or intact, directly on ROS production using original tests, potato protein being the most effective.

## 4. Conclusions

In this study, 10 plant-based protein samples—four grades of pea protein (PeaP1, PeaP2, PeaP3, and PeaP4) and a hydrolyzed pea protein (HPeaP), two grades of wheat protein (WP1 and WP2), and potato, fava bean, and oat proteins (PP, FBP, and OP)—were submitted to SGID. Whey protein (WhP) was used as an animal-sourced protein reference. Undigested and digested protein samples were then tested in vitro to investigate their ability to modulate food intake and glucose homeostasis through CCK and GLP-1 secretion, DPP-IV activity, and opioid receptor binding. They were also evaluated for their ACE-inhibitory activity but also their anti-inflammatory and antioxidant properties. The best candidates answering globally to the main activities explored were PeaP1, PeaP2, HPeaP, PP, and FBP with some specificities (Table 10). Natural peptides could offer safer alternatives to some drugs, either in a curative or in a preventive context like functional foods and personalized nutrition. Nevertheless, further exploration and in vivo validation are needed to investigate whether these protein samples could be potentially valorized in the future in the context of obesity, T2DM, and related cardiovascular risk prevention, or for their antioxidant properties in the context of cardiovascular diseases or aging.

The present study also allows the screening and comparison of the effects of different digested plant-based proteins on numerous bioactivities, using the same tests and in the same conditions. These tests present some limitations, but this study allows us to characterize numerous proteins based on their bioactivities and their health potential and not only based on their amino acid composition and digestibility. Then, this study demonstrates that the SGID of different protein samples generates peptides with different sizes, sequences, and structures. These differences of peptide populations lead to differences in terms of bioactivities. Furthermore, the protein obtention process seems to play a role, since differences for some bioactivities could be observed between the four grades of pea protein (PeaP1, PeaP2, PeaP3, and PeaP4) before and after SGID.

## Figures and Tables

**Table 1:** Description and characterization of the 11 protein samples studied.

Name	Description	% Dry Matter	% Protein
PeaP1	Pea protein—grade 1	93.0	79.1
PeaP2	Pea protein—grade 2	95.2	79.7
PeaP3	Pea protein—grade 3	94.4	80.8
PeaP4	Pea protein—grade 4	95.6	81.5
HPeaP	Hydrolyzed pea protein	94.6	78.3
WP1	Wheat protein—grade 1	94.8	83.7
WP2	Wheat protein—grade 2	93.2	93.2
PP	Potato protein	93.8	79.1
FBP	Fava bean protein	96.0	89.8
OP	Oat protein	96.0	88.3
WhP	Whey protein	95.0	85.5

PeaP1–PeaP4 (4 grades of pea protein), HPeaP (hydrolyzed pea protein), WP1–WP2 (2 grades of wheat protein), PP (potato protein), FBP (fava bean protein), OP (oat protein) and WhP (Whey protein).

**Table 2:** Chemical composition, protease concentration, and pH used for the different compartments of the in vitro simulated gastrointestinal digestion (SGID).

Characteristics	Saliva	Gastric Juice	Duodenal Juice	Bile Juice
Chemical Composition	KCl (12 mM)	KCl (11 mM)	KCl (7.6 mM)	KCl (5 mM)
	KSCN (2 mM)	NaH_2_PO_4_ (2.2 mM)	KH_2_PO_4_ (0.6 mM)	NaCl (90 mM)
	NaH_2_PO_4_ (7.4 mM)	NH_4_Cl (5.7 mM)	NaCl (120 mM)	NaHCO_3_ (69 mM)
	Na_2_SO4 (4 mM)	NaCl (47 mM)	NaHCO_3_ (40 mM)	HCl (1.5 mM)
	NaCl (5 mM)	HCl (6.5 mM)	HCl (1.8 mM)	CO(NH_2_)_2_ (4 mM)
	NaHCO3 (20 mM)	CaCl_2_ (2.7 mM)	MgCl_2_ (0.5 mM)	
	CO(NH_2_)_2_ (3.3 mM)	CO(NH_2_)_2_ (1.4 mM)	CO(NH_2_)_2_ (1.7 mM)	
Proteases		Pepsin 1/40 (*w*/*w*)	Pancreatin 1/50 (*w*/*w*)	
pH	6.8 ± 0.2	1.3 ± 0.2	8.1 ± 0.2	8.2 ± 0.2

**Table 3:** Solubility degree (%) of the 11 protein samples, at each step of the SGID.

Name	Salivary Sample	Gastric Digestion	Intestinal Digestion
PeaP1	20.53	61.65	85.97
PeaP2	43.55	58.67	83.94
PeaP3	47.27	69.47	86.32
PeaP4	100.00	82.81	86.96
HPeaP	81.16	82.39	95.61
WP1	63.33	75.56	93.40
WP2	90.08	100.00	100.00
PP	9.13	51.79	69.77
FBP	39.41	56.72	90.34
OP	18.33	69.35	83.58
WhP	91.97	69.97	100.00

PeaP1–PeaP4 (4 grades of pea protein), HPeaP (hydrolyzed pea protein), WP1–WP2 (2 grades of wheat protein), PP (potato protein), FBP (fava bean protein), OP (oat protein) and WhP (Whey protein).

**Table 4:** Inhibitory concentration inducing 50% DPP-IV activity inhibition (IC_50_) of the 11 protein samples before (salivary sample), during (gastric digestion), and after SGID (intestinal digestion).

DPP-IV-IC_50_ (mg mL^−1^)	Salivary Sample	Gastric Digestion	Intestinal Digestion
PeaP1	ND	1.13 ± 0.05 ^CD^	1.07 ± 0.06 ^bcde^
PeaP2	3.87 ± 1.01	0.85 ± 0.19 ^CD^	0.98 ± 0.55 ^cde^
PeaP3	ND	1.45 ± 0.15 ^BCD^	1.19 ± 0.05 ^bcd^
PeaP4	ND	2.34 ± 1.18 ^AB^	1.46 ± 0.17 ^bc^
HPeaP	1.19 ± 0.12	0.86 ± 0.08 ^CD^	0.79 ± 0.08 ^de^
WP1	ND	2.96 ± 0.34 ^A^	1.64 ± 0.07 ^ab^
WP2	ND	2.96 ± 0.51 ^A^	2.03 ± 0.30 ^a^
PP	ND	1.88 ± 0.21 ^ABC^	1.07 ± 0.12 ^bcde^
FBP	ND	0.65 ± 0.07 ^D^	0.54 ± 0.03 ^e^
OP	ND	0.75 ± 0.07 ^CD^	0.83 ± 0.10 ^cde^
WhP	ND	1.70 ± 0.10 ^BCD^	1.02 ± 0.14 ^bcde^

Values are reported as the mean from triplicate determinations with SD. “ND” indicates the IC_50_ value was too high to be evaluated using this test. Values not sharing any common superscript letter are significantly different according to one-way ANOVA procedure followed by Tukey’s test (intestinal digestions were compared together (a–e, small letters) and gastric digestions together (A–D, capital letters)). PeaP1–PeaP4 (4 grades of pea protein), HPeaP (hydrolyzed pea protein), WP1–WP2 (2 grades of wheat protein), PP (potato protein), FBP (fava bean protein), OP (oat protein) and WhP (Whey protein).

**Table 5:** ED_50_ values expressed in mg mL^−1^ (dry matter) for opioid receptor binding for each of the 11 protein samples before (salivary sample) and after SGID (intestinal digestion).

Opioid R-ED_50_ (mg mL^−1^)	Salivary Sample	Intestinal Digestion
PeaP1	26.80	14.90
PeaP2	4.98	4.23
PeaP3	4.63	2.44
PeaP4	24.98	3.17
HPeaP	8.60	10.27
WP1	39.50	26.73
WP2	80.30	17.90
PP	14.55	15.40
FBP	2.83	12.51
OP	31.03	10.25
WhP	78.21	22.70

PeaP1–PeaP4 (4 grades of pea protein), HPeaP (hydrolyzed pea protein), WP1–WP2 (2 grades of wheat protein), PP (potato protein), FBP (fava bean protein), OP (oat protein) and WhP (Whey protein).

**Table 6:** Inhibitory concentration inducing 50% ACE activity inhibition (IC_50_) of the 11 protein samples before (salivary sample), during (gastric digestion), and after SGID (intestinal digestion).

ACE-IC_50_ (µg mL^−1^)	Salivary Sample	Gastric Digestion	Intestinal Digestion
PeaP1	ND	90.70 ± 25.80 ^CD^	101.19 ± 23.15 ^cd^
PeaP2	407.78 ± 28.10	37.37 ± 19.83 ^D^	221.79 ± 45.57 ^ab^
PeaP3	ND	47.38 ± 44.33 ^CD^	185.54 ± 9.97 ^abc^
PeaP4	ND	187.97 ± 45.38 ^AB^	29.54 ± 34.98 ^d^
HPeaP	148.97 ± 40.79	93.37 ± 24.74 ^CD^	113.64 ± 50.51 ^cd^
WP1	ND	60.79 ± 12.22 ^CD^	63.82 ± 20.18 ^d^
WP2	ND	186.78 ± 0.91 ^AB^	265.46 ± 22.88 ^a^
PP	ND	107.34 ± 40.79 ^BCD^	85.23 ± 19.24 ^d^
FBP	ND	133.50 ± 35.09 ^BC^	52.02 ± 43.06 ^d^
OP	ND	98.04 ± 32.21 ^CD^	99.46 ± 9.88 ^cd^
WhP	ND	126.38 ± 82.34 ^BCD^	127.50 ± 25.61 ^bcd^

Values are reported as the mean from triplicate determinations with SD. “ND” indicates the IC_50_ value was too high to be evaluated using this test. Values not sharing any common superscript letter are significantly different according to one-way ANOVA procedure followed by Tukey’s test (intestinal digestions were compared together (a–d, small letters) and gastric digestions together (A–D, capital letters)). PeaP1–PeaP4 (4 grades of pea protein), HPeaP (hydrolyzed pea protein), WP1–WP2 (2 grades of wheat protein), PP (potato protein), FBP (fava bean protein), OP (oat protein) and WhP (Whey protein).

**Table 7:** Inhibitory concentration inducing 50% O_2_^−^ production inhibition (IC_50_) of the 11 protein samples before (salivary sample) and after SGID (intestinal digestion).

O_2_^−^-IC_50_ (mg mL^−1^)	Salivary Sample	Intestinal Digestion
PeaP1	0.70	ND
PeaP2	ND	ND
PeaP3	ND	ND
PeaP4	ND	ND
HPeaP	ND	ND
WP1	ND	ND
WP2	1.30	ND
PP	2.00	1.80
FBP	ND	ND
OP	2.50	ND
WhP	ND	ND

Values are reported as the mean from triplicate determinations. IC_50_ values are expressed in mg mL^−1^ (dry matter). IC_50_ values, which could not be determined (higher than 5 mg mL^−1^), are represented by “ND”. PeaP1–PeaP4 (4 grades of pea protein), HPeaP (hydrolyzed pea protein), WP1–WP2 (2 grades of wheat protein), PP (potato protein), FBP (fava bean protein), OP (oat protein) and WhP (Whey protein).

**Table 8:** Inhibitory concentration inducing 50% H_2_O_2_ production inhibition (IC_50_) of the 11 protein samples before (salivary sample) and after SGID (intestinal digestion).

H_2_O_2_-IC_50_ (mg mL^−1^)	Salivary Sample	Intestinal Digestion
PeaP1	0.65	0.55
PeaP2	0.65	1.40
PeaP3	0.80	4.60
PeaP4	1.05	ND
HPeaP	0.65	1.20
WP1	ND	ND
WP2	2.10	ND
PP	0.65	1.20
FBP	0.65	0.65
OP	0.75	3.05
WhP	4.45	2.08

Values are reported as the mean from triplicate determinations. IC_50_ values are expressed in mg mL^−1^ (dry matter). IC_50_ values which could not be determined (higher than 5 mg.mL^−1^) are represented by “ND”. PeaP1–PeaP4 (4 grades of pea protein), HPeaP (hydrolyzed pea protein), WP1–WP2 (2 grades of wheat protein), PP (potato protein), FBP (fava bean protein), OP (oat protein) and WhP (Whey protein).

**Table 9:** Inhibitory concentration inducing 50% HO^.^ production inhibition (IC_50_) of the 11 protein samples before (salivary sample) and after SGID (intestinal digestion).

HO-IC_50_ (mg mL^−1^)	Salivary Sample	Intestinal Digestion
PeaP1	1.60	2.05
PeaP2	1.25	1.00
PeaP3	1.10	1.55
PeaP4	1.60	1.35
HPeaP	1.45	2.50
WP1	4.50	1.65
WP2	1.35	0.85
PP	1.00	1.75
FBP	1.30	1.45
OP	1.50	1.70
WhP	2.95	1.33

Values are reported as the mean from triplicate determinations. IC_50_ values are expressed in mg mL^−1^ (dry matter). PeaP1–PeaP4 (4 grades of pea protein), HPeaP (hydrolyzed pea protein), WP1–WP2 (2 grades of wheat protein), PP (potato protein), FBP (fava bean protein), OP (oat protein) and WhP (Whey protein).

**Table 10:** Bioactivity scores of the 11 protein samples.

Protein	Metabolic Activity				Anti-Hypertensive	Anti-Inflammatory	Antioxidant			SCORE
	CCK	GLP-1	DPP-IV	OR	ACE	IL-8	O_2_^−^	H_2_O_2_	HO^.^	
PeaP1	1	1	2	1	2	0	0	2	1	10
PeaP2	1	2	2	2	1	0	0	2	1	11
PeaP3	1	1	1	2	1	0	0	1	1	8
PeaP4	1	1	1	2	2	0	0	0	1	8
HPeaP	1	1	2	1	2	0	0	2	1	10
WP1	1	1	1	1	2	0	0	0	1	7
WP2	1	1	1	1	1	0	0	0	1	6
PP	2	2	2	1	2	0	2	2	1	14
FBP	1	1	2	1	2	0	0	2	1	10
OP	1	1	2	1	2	0	0	1	1	9
WhP	1	1	2	1	2	0	0	1	1	9

“0”—no activity detected; “1”—activity detected; “2”—good activity detected. OR—opioid receptor.

## Supplementary Materials

The following are available online at https://www.mdpi.com/2072-6643/12/12/3746/s1, Figure S1: Apparent molecular weight peptide profiles of PeaP1 at the end of the SGID intestinal step, obtained after SEC-FPLC, Figure S2: Apparent molecular weight peptide profiles of PeaP2 at the end of the SGID intestinal step, obtained after SEC-FPLC, Figure S3: Apparent molecular weight peptide profiles of PeaP3 at the end of the SGID intestinal step, obtained after SEC-FPLC, Figure S4: Apparent molecular weight peptide profiles of PeaP4 at the end of the SGID intestinal step, obtained after SEC-FPLC, Figure S5: Apparent molecular weight peptide profiles of HPeaP at the end of the SGID intestinal step, obtained after SEC-FPLC, Figure S6: Apparent molecular weight peptide profiles of WP1 at the end of the SGID intestinal step, obtained after SEC-FPLC, Figure S7: Apparent molecular weight peptide profiles of WP2 at the end of the SGID intestinal step, obtained after SEC-FPLC, Figure S8: Apparent molecular weight peptide profiles of PP at the end of the SGID intestinal step, obtained after SEC-FPLC, Figure S9: Apparent molecular weight peptide profiles of FBP at the end of the SGID intestinal step, obtained after SEC-FPLC, Figure S10: Apparent molecular weight peptide profiles of OP at the end of the SGID intestinal step, obtained after SEC-FPLC, Figure S11: Apparent molecular weight peptide profiles of WhP at the end of the SGID intestinal step, obtained after SEC-FPLC, Figure S12: Apparent molecular weight peptide profiles of Blank SGID at the end of the SGID intestinal step, obtained after SEC-FPLC.
