# Supplementary material for: Explorative Screening of Bioactivities Generated by Plant-Based Proteins after In Vitro Static Gastrointestinal Digestion
Source: Nutrients. 2020 Dec 5;12(12):3746. doi: 10.3390/nu12123746 (PMC7762166; doi:10.3390/nu12123746)
Supplement: Supplementary file 1 [file nutrients-12-03746-s001.pdf]

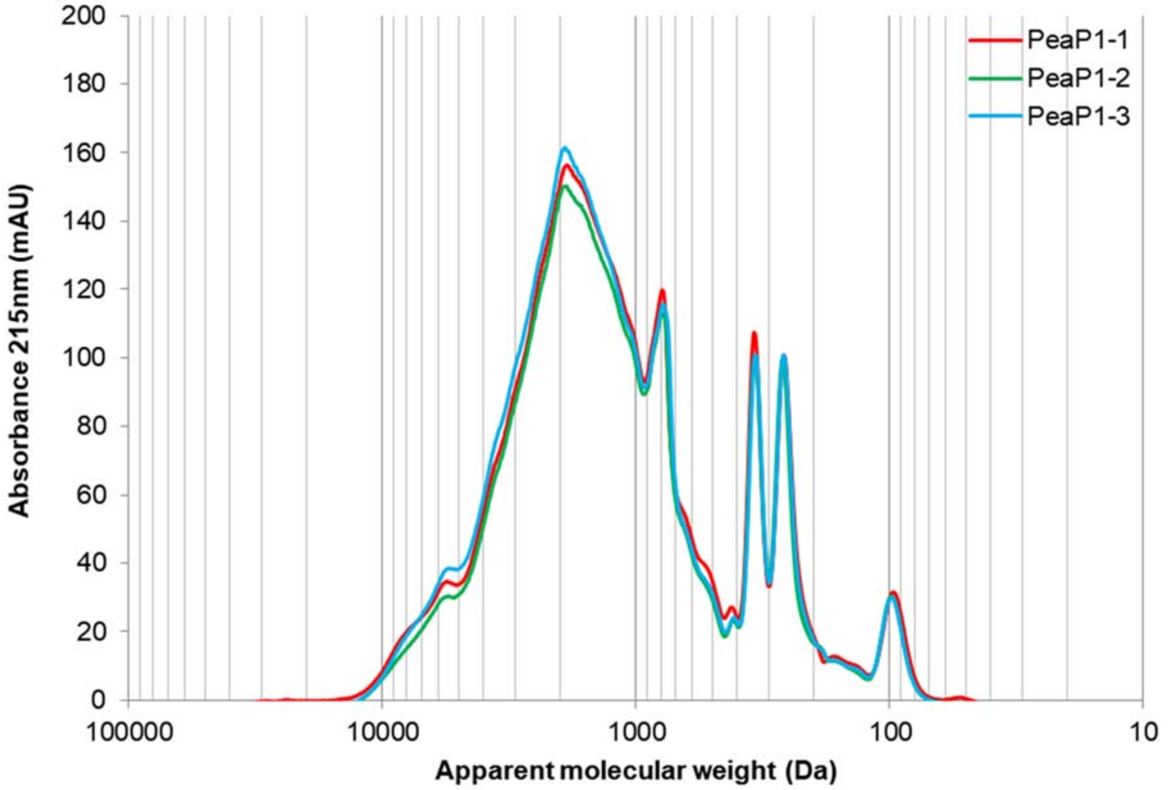

Figure S1. Apparent molecular weight peptide profiles of PeaP1 at the end of the SGID intestinal step, obtained after SEC-FPLC. Each curve represents the peptide profile of one replicate.

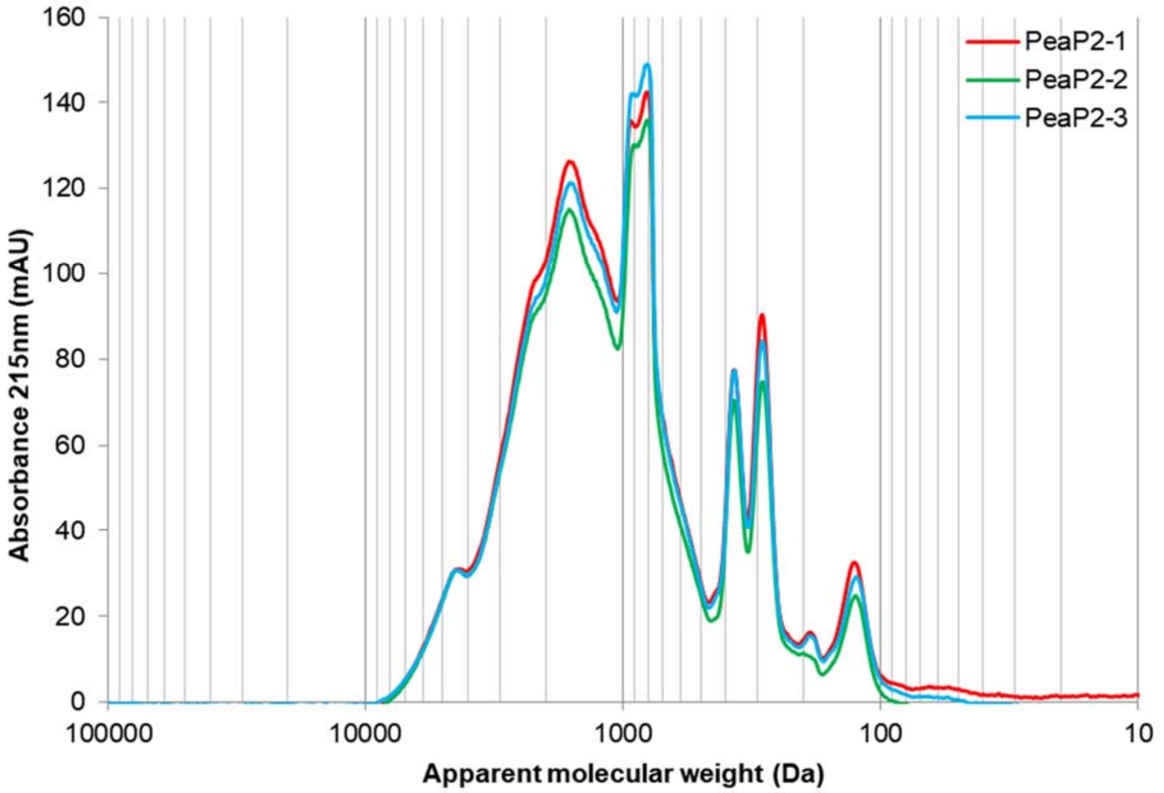

Figure S2. Apparent molecular weight peptide profiles of PeaP2 at the end of the SGID intestinal step, obtained after SEC-FPLC. Each curve represents the peptide profile of one replicate.

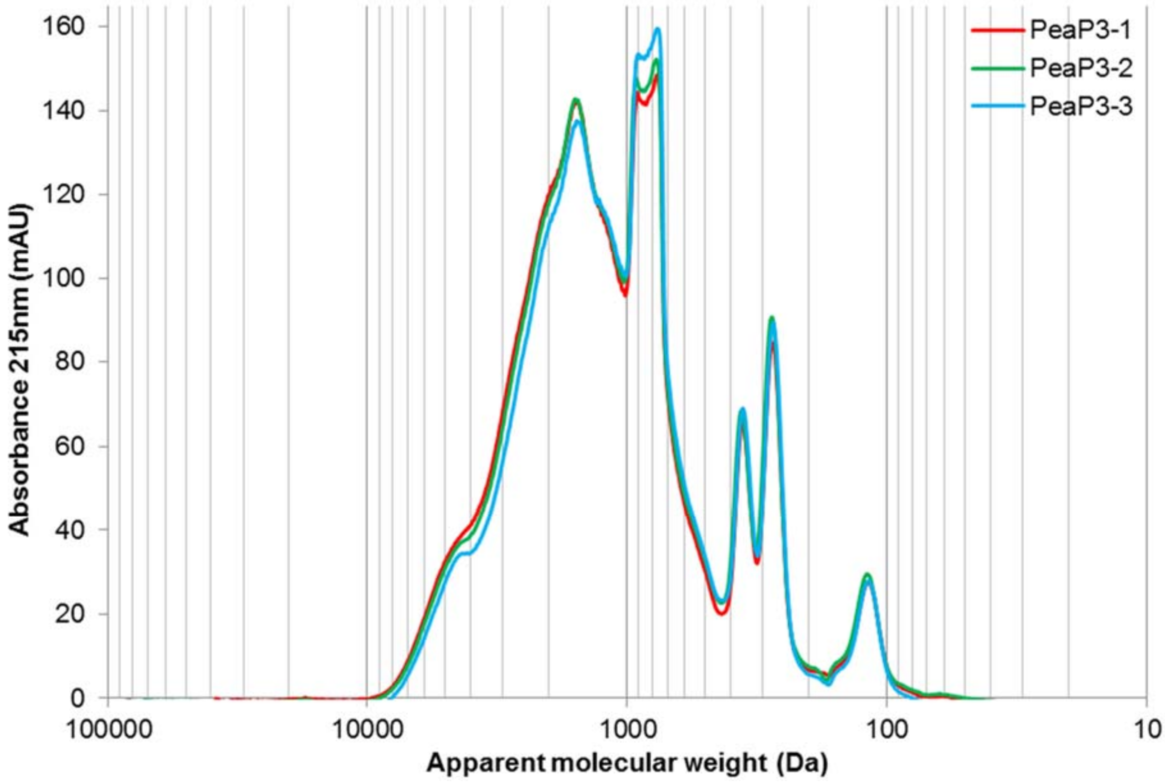

Figure S3. Apparent molecular weight peptide profiles of PeaP3 at the end of the SGID intestinal step, obtained after SEC-FPLC. Each curve represents the peptide profile of one replicate.

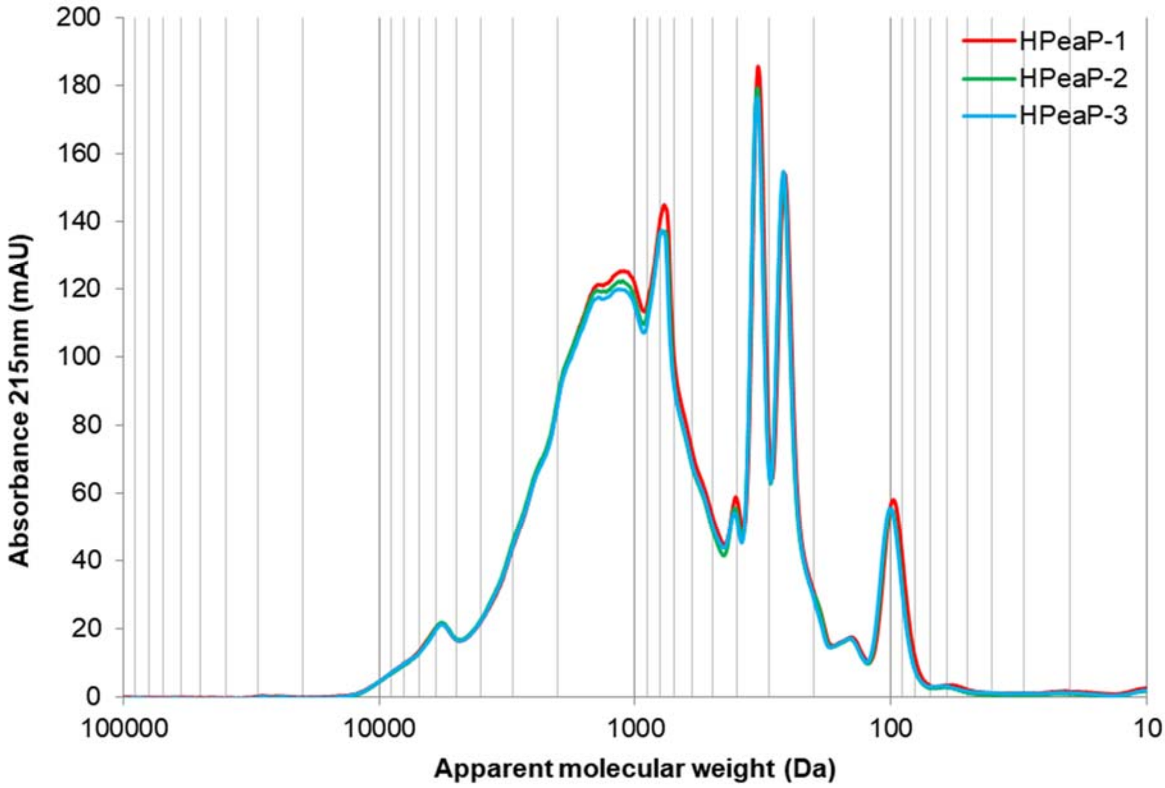

Figure S4. Apparent molecular weight peptide profiles of HPeaP at the end of the SGID intestinal step, obtained after SEC-FPLC. Each curve represents the peptide profile of one replicate.

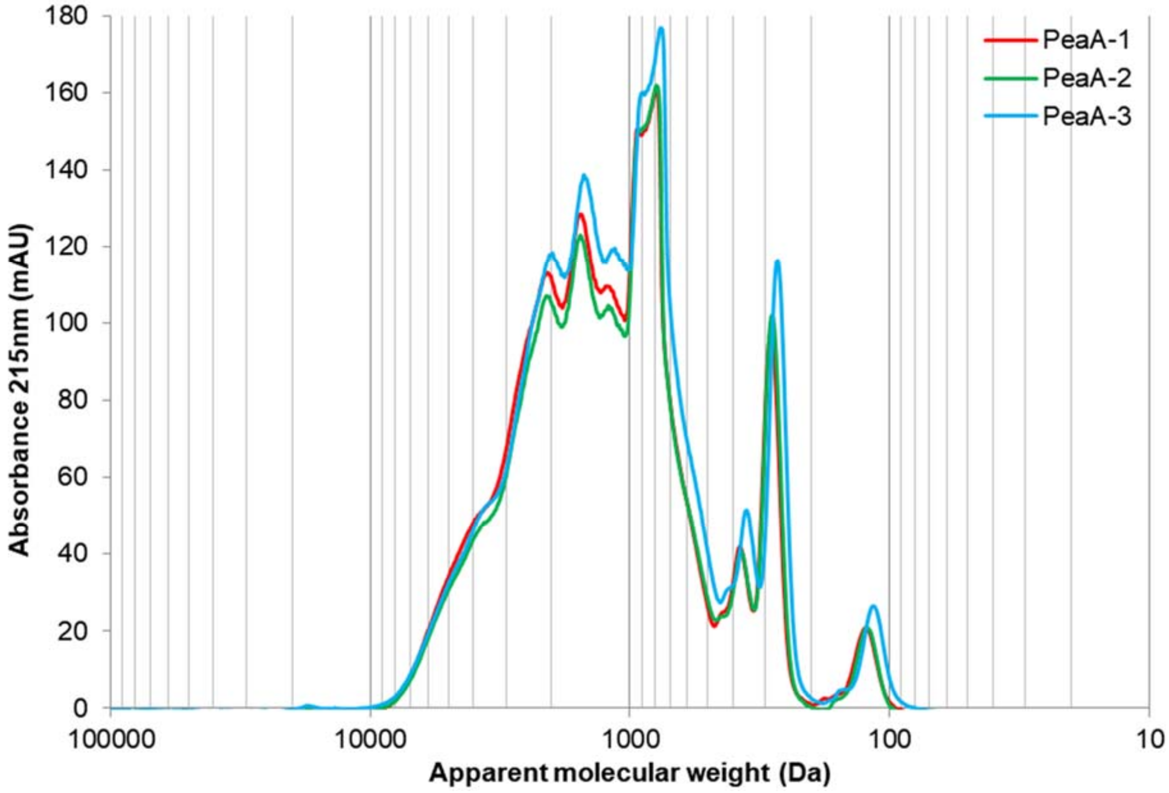

Figure S5. Apparent molecular weight peptide profiles of PeaA at the end of the SGID intestinal step, obtained after SEC-FPLC. Each curve represents the peptide profile of one replicate.

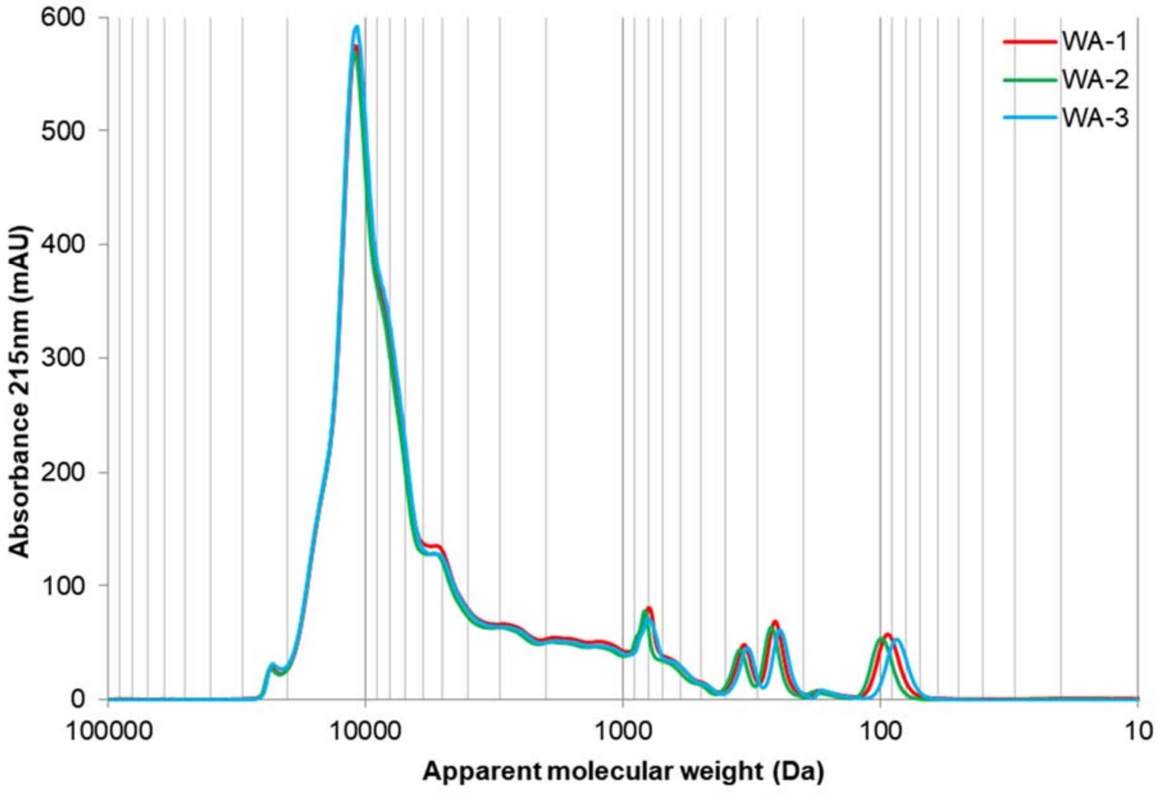

Figure S6. Apparent molecular weight peptide profiles of WA at the end of the SGID intestinal step, obtained after SEC-FPLC. Each curve represents the peptide profile of one replicate.

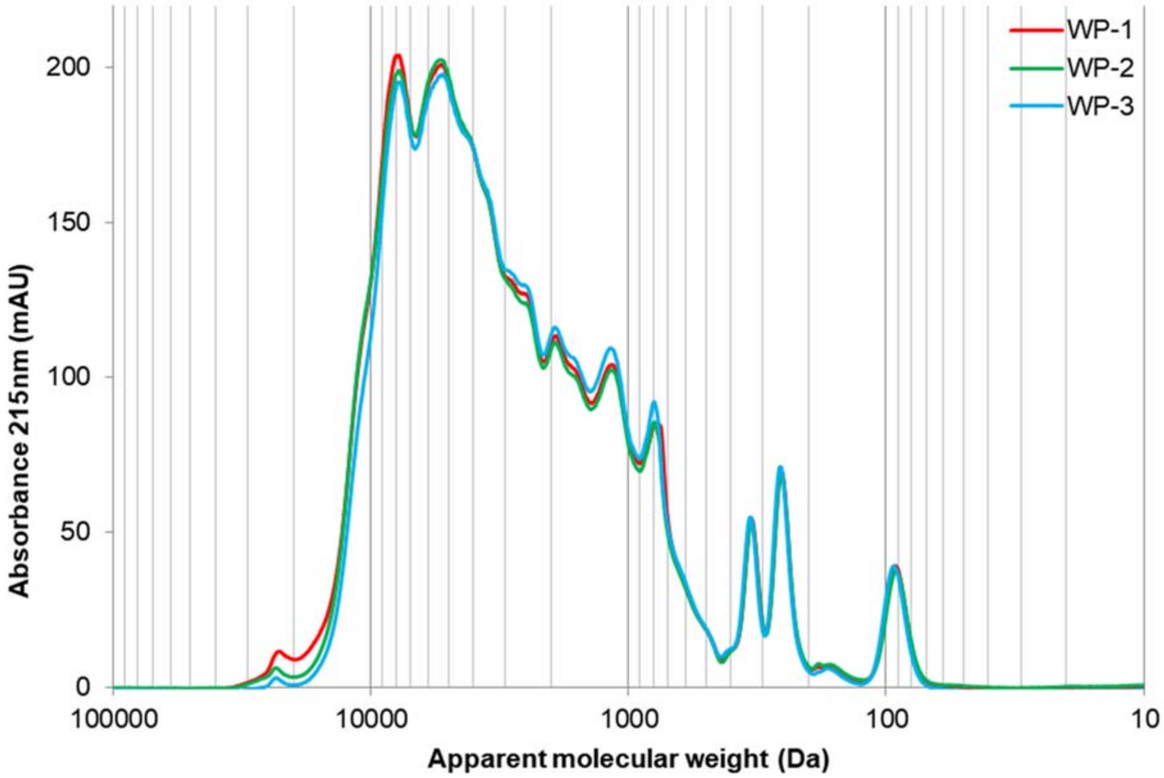

Figure S7. Apparent molecular weight peptide profiles of WP at the end of the SGID intestinal step, obtained after SEC-FPLC. Each curve represents the peptide profile of one replicate.

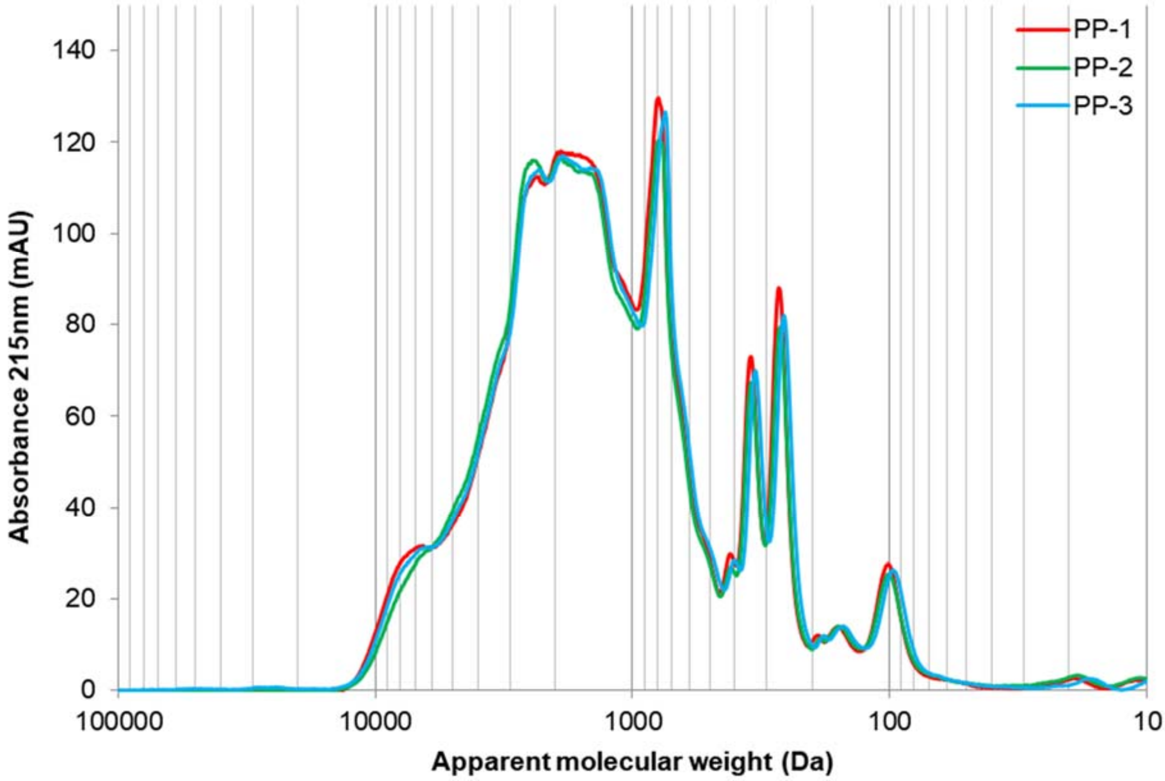

Figure S8. Apparent molecular weight peptide profiles of PP at the end of the SGID intestinal step, obtained after SEC-FPLC. Each curve represents the peptide profile of one replicate.

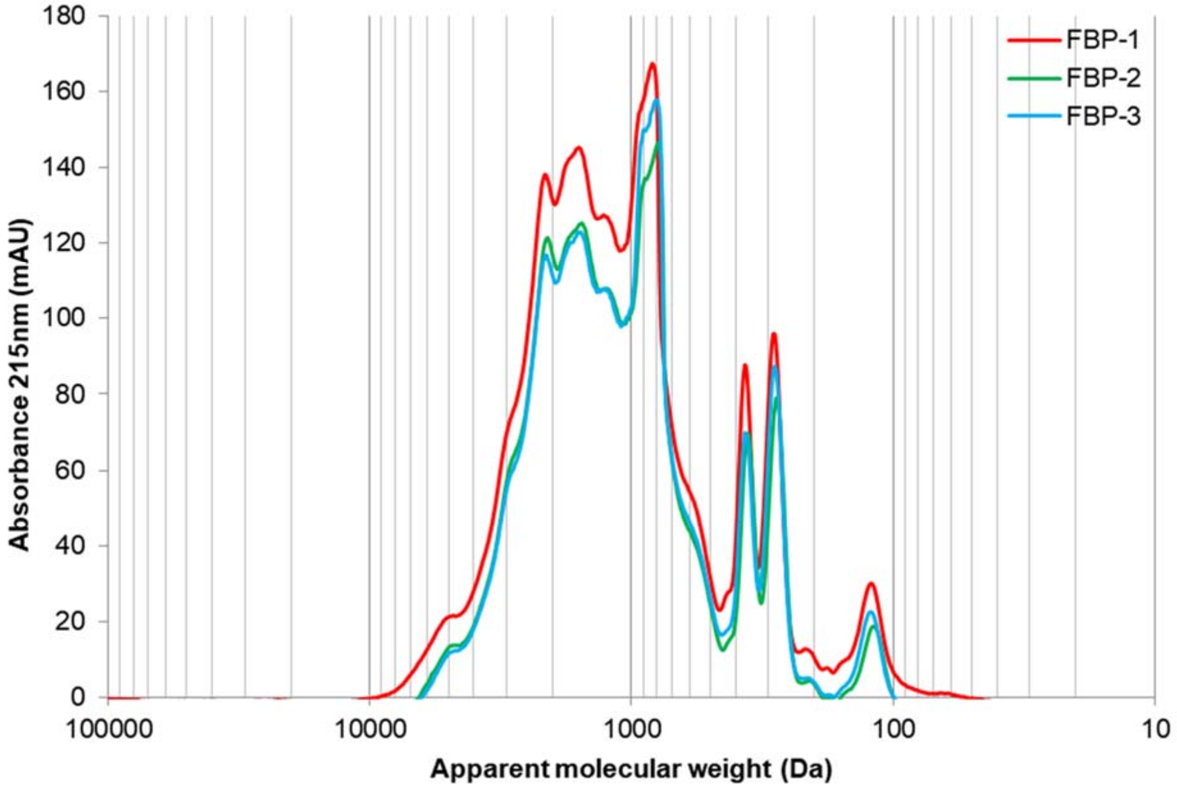

Figure S9. Apparent molecular weight peptide profiles of FBP at the end of the SGID intestinal step, obtained after SEC-FPLC. Each curve represents the peptide profile of one replicate.

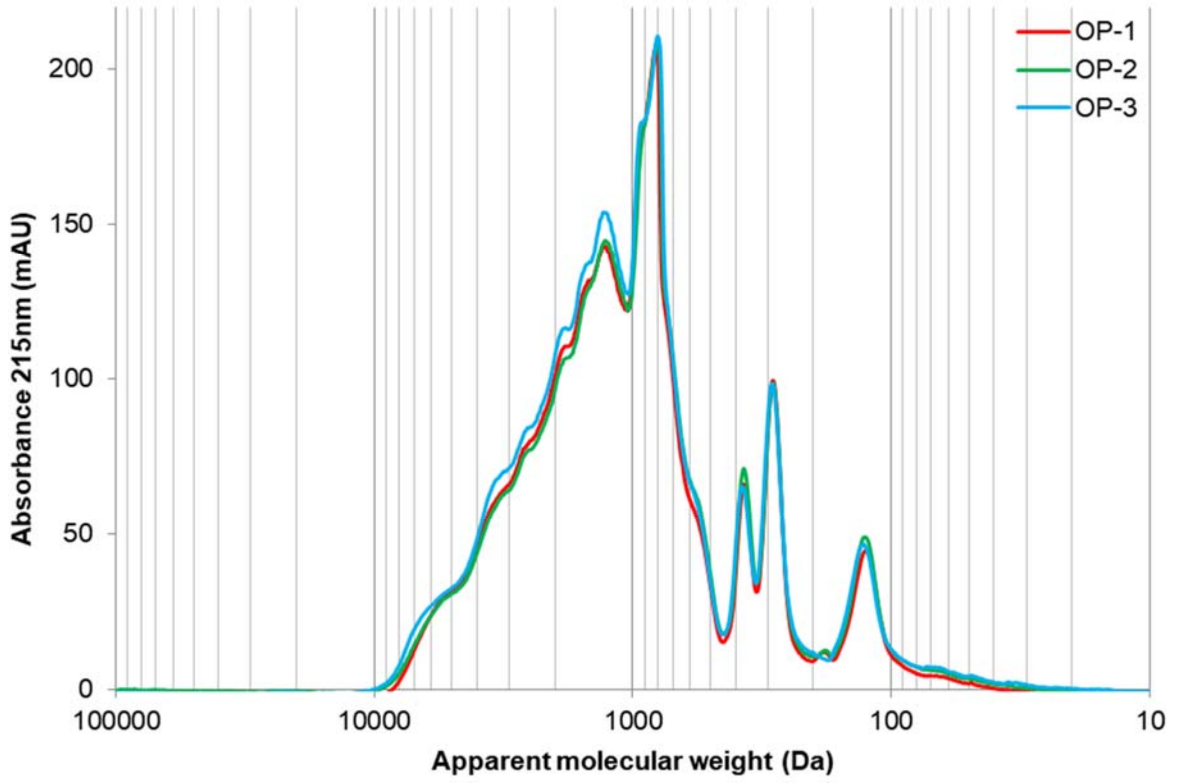

Figure S10. Apparent molecular weight peptide profiles of OP at the end of the SGID intestinal step, obtained after SEC-FPLC. Each curve represents the peptide profile of one replicate.

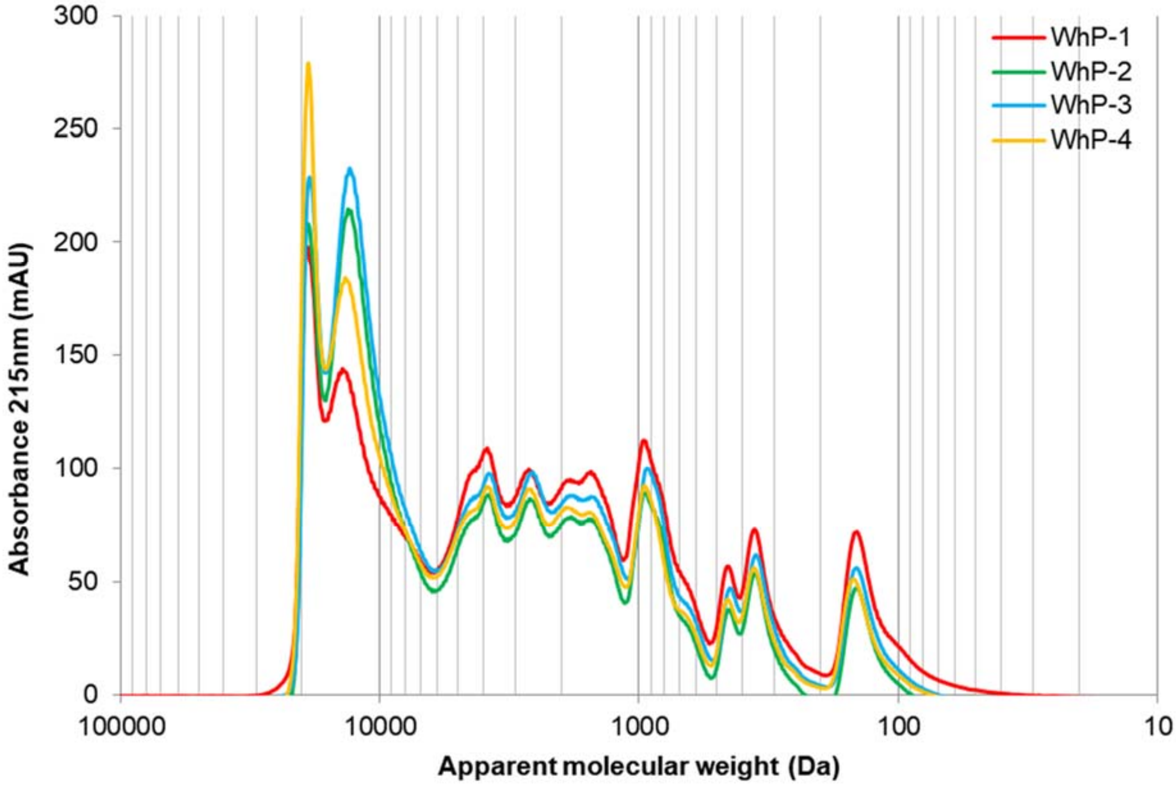

Figure S11. Apparent molecular weight peptide profiles of WhP at the end of the SGID intestinal step, obtained after SEC-FPLC. Each curve represents the peptide profile of one replicate.

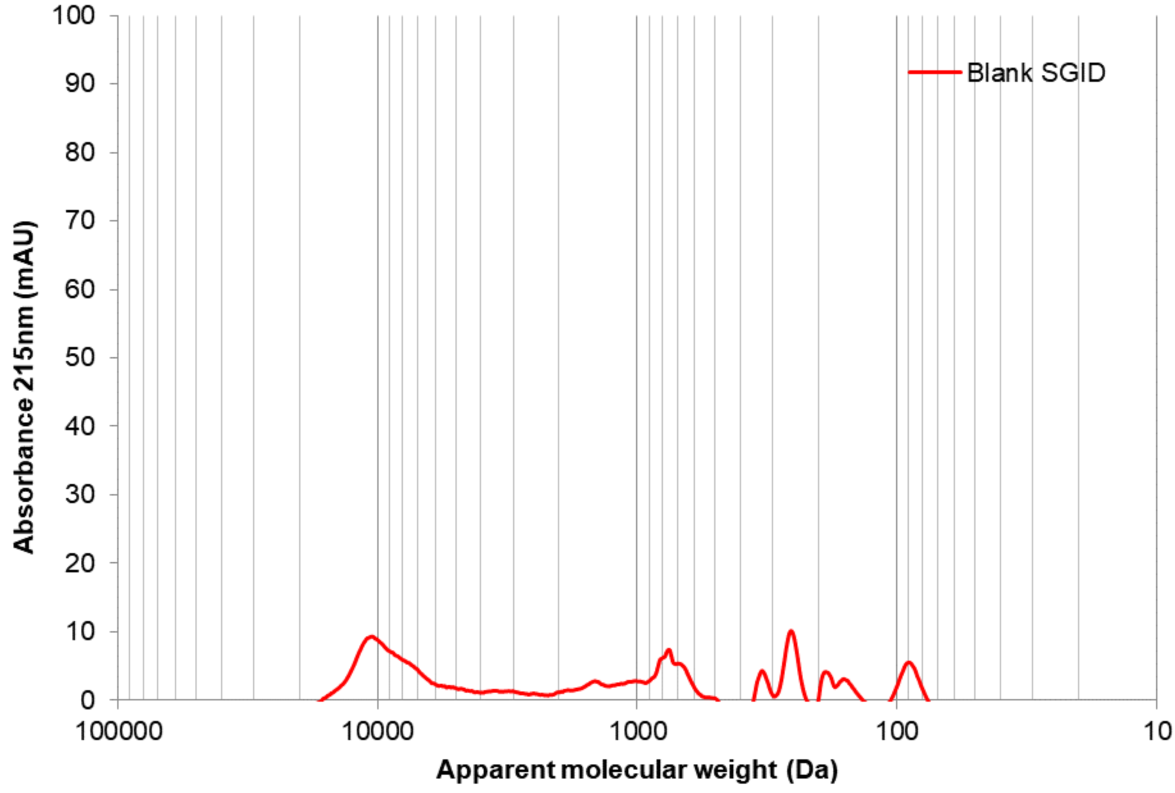

Figure S12. Apparent molecular weight peptide profiles of Blank SGID at the end of the intestinal step, obtained after SEC-FPLC.
